# Crystal structure of the insecticide ethiprole (C_13_H_9_Cl_2_F_3_N_4_OS): a case study of whole-mol­ecule configurational disorder

**DOI:** 10.1107/S205698902300035X

**Published:** 2023-01-19

**Authors:** Vinaya, Yeriyur B. Basavaraju, Gejjelegere R. Srinivasa, Mellekatte T. Shreenivas, Hemmige S. Yathirajan, Sean Parkin

**Affiliations:** a Department of Studies in Chemistry, University of Mysore, Manasagangotri, Mysuru-570 006, India; b Honeychem Pharma Research Pvt. Ltd., Peenya Industrial Area, Bengaluru-560 058, India; c Department of Chemistry, University of Kentucky, Lexington, KY, 40506-0055, USA; Universidad de Los Andes Mérida, Venezuela

**Keywords:** ethiprole, phenyl­pyrazole insecticide, whole-mol­ecule disorder, configurational disorder, refinement strategy, instructional tool, crystal structure

## Abstract

The crystal structure of ethiprole {systematic name: 5-amino-1-[2,6-di­chloro-4-(tri­fluoro­meth­yl)phen­yl]-4-ethane­sulfinyl-1*H*-imidazole-3-carbo­nitrile}, C_13_H_9_Cl_2_F_3_N_4_OS, a phenyl­pyrazole-based insecticide, is presented. The pyrazole ring carries four substituents: an N-bound 2,6-di­chloro-4-tri­fluoro­methyl­phenyl ring and C-bound amine, ethane­sulfinyl, and cyano groups. The sulfur atom of the ethane­sulfinyl group is trigonal–pyramidal and stereogenic. The structure exhibits whole-mol­ecule configurational disorder due to superposition of enanti­omers. The crystal packing is dominated by strong N—H⋯O and N—H⋯N hydrogen bonds, which form *R* ^4^_4_(18) and *R* ^2^_2_(12) ring motifs. Since the ethiprole mol­ecule is quite small, and structure solution and refinement were straightforward, the structure presents a convenient instructional example for modelling whole-body disorder of a non-rigid mol­ecule. To this end, a step-by-step overview of the model-building and refinement process is also given. The structure could form the basis of a useful classroom, practical, or workshop-style example.

## Abstract 2

The crystal structure of the phenyl­pyrazole insecticide ethiprole is presented along with a step-by-step overview of the model building and refinement process.

## Chemical context

Ethiprole, systematic name 5-amino-1-[2,6-di­chloro-4-(tri­fluoro­meth­yl)phen­yl]-4-(ethane­sulfin­yl)-1*H*-pyrazole-3-carb­o­­nitrile (C_13_H_9_Cl_2_F_3_N_4_OS), is a phenyl­pyrazole-based insecticide. This class of compounds target an insect’s central nervous system, making it toxic to the host by blocking the glutamate-gated chloride channel. They are effective against a broad spectrum of chewing and sucking insects, showing pronounced plant systemic activity (Wu, 1998 ▸), as well as offering protection against stored-grain insect pests (Arthur, 2002 ▸). Fipronil and fipronil sulfone are related insecticides. The design, synthesis, and mode of action of phenyl­pyrazoles containing the 2,2,2-tri­chloro-1-alk­oxy­ethyl functionality has been published by Zhao *et al.* (2010 ▸). The synthesis and pharmacological activities of pyrazole derivatives were reviewed by Karrouchi *et al.* (2018 ▸), and advances in their synthesis were described by Fustero *et al.* (2011 ▸). Further developments in the synthesis and biological evaluations of pyrazole derivatives were reviewed recently by Ebenezer *et al.* (2022 ▸). In light of the general structure–function relationships of phenyl­pyrazole insecticides, it is surprising that the crystal structure of ethiprole has not previously been published. One possible reason could be the presence of whole-mol­ecule disorder (*vide infra*), which provided further impetus for our crystallographic study of ethiprole. 
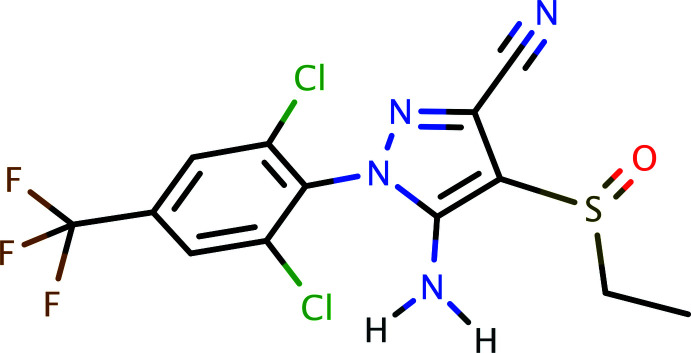


Ethiprole is a chiral mol­ecule by virtue of the trigonal–pyramidal geometry at the sulfur atom of its ethane­sulfinyl group. Commercial formulations are, however, racemic. The crystal structure presented here is centrosymmetric, but was found to incorporate configurational whole-mol­ecule disorder. The phenomenon of whole-mol­ecule disorder is not new; well-known examples include azulene (Robertson *et al.*, 1962 ▸) and uric acid dihydrate (Parkin & Hope, 1998 ▸), amongst many others. Unlike ethiprole, both azulene and uric acid are rigid planar mol­ecules. In azulene, disorder results from the mol­ecule (which lacks inversion point symmetry) being situated on a crystallographic inversion centre (and is therefore disordered exactly 50:50), while in uric acid dihydrate the minor disorder component results from a non-crystallographic 180° flip of the mol­ecule, which fortuitously happens to remain compatible with the hydrogen-bonding environment of its major-occupancy counterpart. In ethiprole, however, the mol­ecule is not rigid; indeed it has several inter­nal degrees of freedom. The disorder results from superposition of enanti­omers, with concomitant torsional relaxation of the other functional groups to satisfy hydrogen-bonding requirements and best fill the available space. Since the ethiprole mol­ecule is quite small, and structure solution and refinement were relatively straightforward, we thought it might serve as a conven­ient instructional example to showcase the concept and treatment of whole-mol­ecule disorder for a non-rigid mol­ecule. To this end, we also present a step-by-step overview of one way to proceed from structure solution through model building and refinement to a chemically and crystallographically sensible final model.

## Structural commentary

**Figure 1:**
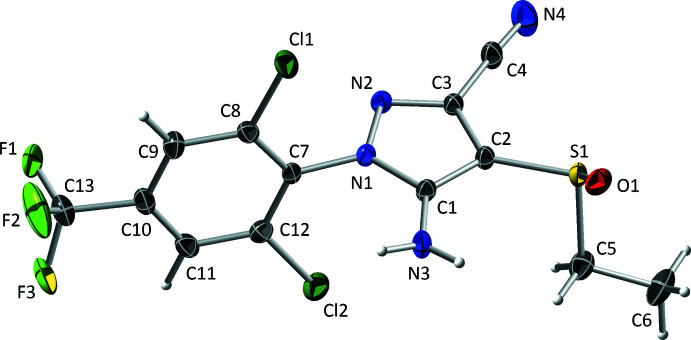
An ellipsoid plot (50% probability) of the major disorder component in crystals of ethiprole. Hydrogen atoms are drawn as spheres of arbitrary radius.

**Figure 2:**
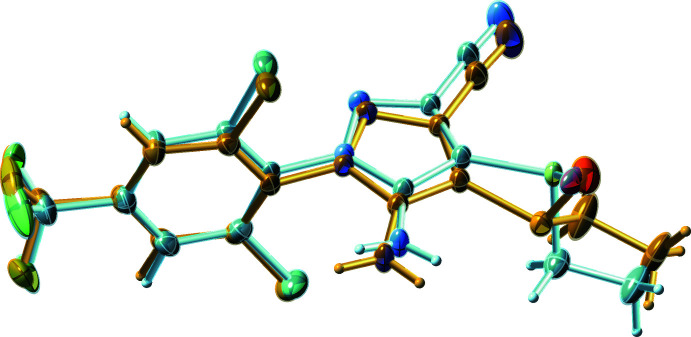
A plot showing the superposition of the minor-disorder component (orange) on the major-disorder component (light blue).

The ethiprole mol­ecule (Fig. 1 ▸) consists of a phenyl­pyrazole backbone with Cl atoms at the 2- and 6-positions of the benzene ring and a CF_3_ group at the 4-position. The pyrazole ring connects to benzene by one of its ring nitro­gen atoms, with dihedral angle 80.4 (2)° for the major component [minor is 79.7 (12)°] and carries an NH_2_ group on the carbon adjacent to the ring-linking nitro­gen. The ethane­sulfinyl substituent is attached to the middle carbon of the pyrazole ring, with a cyano group on the remaining carbon. All bond lengths and angles in ethiprole have normal values, but there is slight deviation of substituents away from the plane of the benzene ring, *i.e.*, C13 by 0.174 (9) Å, N1 by 0.162 (8) Å on one side of the ring and Cl1 by 0.096 (7) Å and Cl2 by 0.078 (12) Å on the other side. The sulfur atom of the ethane­sulfinyl group is trigonal–pyramidal and therefore stereogenic, but since the structure is centrosymmetric, the crystals are, of necessity, racemic. Despite the equal presence of both optical isomers, each asymmetric unit contains disorder components [major and minor fractions are 86.70 (18)% and 13.30 (18)%, respectively] of the opposite hand (*i.e., R* or *S* at the sulfur superimposed on *S* or *R*, respectively), as shown in Fig. 2 ▸. In addition to the dihedral angle mentioned above, inter­nal degrees of freedom in the mol­ecule correspond to torsions about the N1—C7, C10—C13, and C2—S1 bonds, which are summarized for both disorder components in Table 1 ▸. A detailed step-by-step breakdown of one way to build a satisfactory model for the whole-mol­ecule configurational disorder is given in *Section 6: Structure solution and step-by-step refinement overview*.

## Supra­molecular features

**Figure 3:**
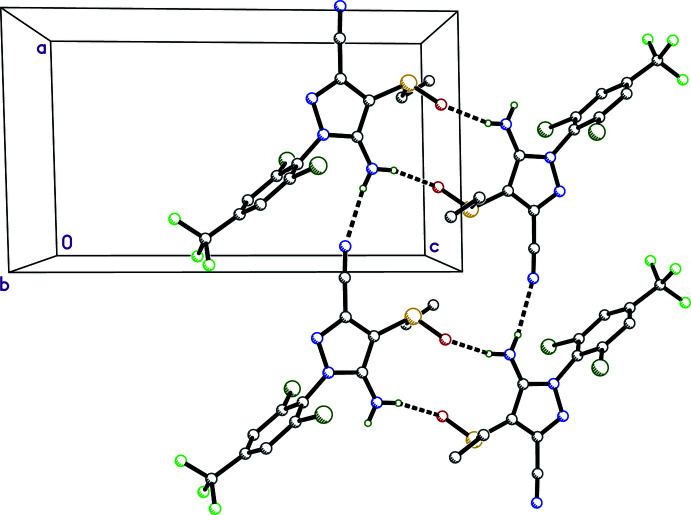
A packing plot viewed down the *b*-axis showing strong hydrogen bonds (dashed lines) that form 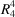 (18) and 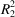 (12) ring motifs. Hydrogen atoms not involved in hydrogen bonding are omitted for the sake of clarity.

Given the relatively small occupancy fraction of the minor disorder component (only ∼13%), detailed description of supra­molecular inter­actions given here is limited to the major component. The proximity of superimposed disorder components (Fig. 2 ▸), however, suggests that the hydrogen-bonding motifs are compatible with both major–minor and minor–minor inter­actions. There are only two strong inter­molecular hydrogen bonds for the major component (Table 2 ▸), and both involve the two hydrogens of the amine group at N3 as donor. These are N3—H3*NA*⋯O1^i^ [*D_D⋯A_* = 2.820 (6) Å] to an inversion-related mol­ecule and N3—H3*NB*⋯N4^ii^ [*D_D⋯A_* = 3.150 (4) Å] involving a mol­ecule adjacent along the *a*-axis direction (symmetry codes are as per Table 2 ▸). In combination with further inversion-related mol­ecules, these hydrogen bonds generate 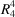 (18) and 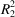 (12) ring motifs (Fig. 3 ▸), which link together to form tapes parallel to the *a*-axis. There are weaker contacts involving C—H as donor included in Table 2 ▸, but of these, only C9—H9⋯O1^iii^ at 3.17 (4) Å is likely to have any structural importance.

## Database and literature survey

There are a large number of structurally and chemically related compounds present in the CSD (CSD version 5.43 with all updates through September 2022; Groom *et al.*, 2016 ▸). A recent paper by Priyanka *et al.* (2022 ▸) on *N*-{3-cyano-1-[2,6-di­chloro-4-(tri­fluoro-meth­yl)phen­yl]-4-(ethyl­sulfan­yl)-1*H*-pyrazol-5-yl}-2,2,2-tri­fluoro­acetamide (CSD entry FERPOL) recorded 82 matches for a 1-phenyl-cyano­pyrazole search fragment. Fine tuning of this search fragment by specifying any N-bound group at C1 reduced the number of matches to 76, while inclusion of 2,6-di­chloro-4-(tri­fluoro­meth­yl)phenyl at N1 gave 60 hits. The requirement of a sulfur-bound group at C2 reduced this to eight unique structures, two of which were dimers. A table of the six best structural matches, plus three closely related compounds was given (see Priyanka *et al.*, 2022 ▸ and references therein). The structure of ethiprole would fit well in that table.

The phenomenon of whole-mol­ecule disorder is not uncommon. A search of the CSD for ‘whole-mol­ecule disorder’, however, gave only 39 hits, but we suspect the true number is higher as not all relevant entries would have been flagged as such in the CSD. A search for ‘configurational disorder’ affecting only whole mol­ecules returned three structures (CUHDOY, CUHDUE, CUHFAM; Bouwstra *et al.*, 1985 ▸), but these involve mixed crystals of *trans*-stilbene and *trans*-azo­benzene. In those three structures the configurations are exclusively *trans*, so the disorder is better described as *orientational* because each disorder component has the same (*i.e.*, *trans*) configuration. One other case is a structure purported to be a monoclinic polymorph of *meso*-(*E*,*E*)-1,10-[1,2-bis­(4-chloro­phen­yl)ethane-1,2-di­yl]-bis­(phenyl­diazene), CSD entry PAGCEI01 (Mohamed *et al.*, 2016 ▸), but the model as presented is severely distorted, including C—C distances as long as 1.695 (7) Å and bond angles in the range 85.9 (4)–139.9 (6)° for ostensibly *sp^3^*-hybridized carbon atoms. The improbable distortions result from inversion symmetry in the assigned space group of type *C*2/*c* (see in particular Fig. 2 ▸ of Mohamed *et al.*, 2016 ▸). A superposition of *S*,*S* and *R*,*R* isomers, with a smaller amount of the *meso* form, is more likely.

## Synthesis and crystallization

**Figure 4:**
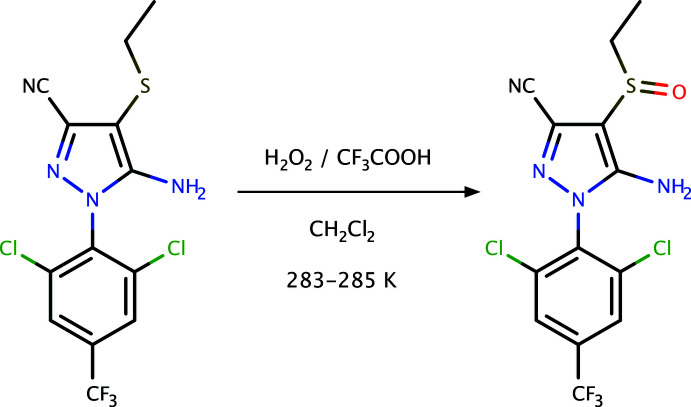
A general reaction scheme for the synthesis of ethiprole.

Tri­fluoro­acetic acid (0.5 mL) was added to a stirred solution of 5-amino-1-[2,6-di­chloro-4-(tri­fluoro­meth­yl)phen­yl]-4-ethyl­thio-1*H*-pyrazole-3-carbo­nitrile (0.19 g, 0.5 mmol) in CH_2_Cl_2_ (2.5 mL) at 283–285 K. Hydrogen peroxide (0.1 mL of 30%, *w*/*w*) was added over 20 min. while maintaining the temperature at 283–285 K and the mixture was kept at the same temperature for a further 3 h. Then, CH_2_Cl_2_ (5 mL) was added followed by sodium hydrogen sulfite to quench any remaining hydrogen peroxide, and the mixture was maintained below 288 K for 20 min. Water (10 mL) was then added, and the mixture was subjected to a careful extraction with a portion of CH_2_Cl_2_ (50 mL). The organic phase was separated off and dried over anhydrous MgSO_4_ and the volatile substances were removed under reduced pressure. The residue was subjected to chromatography on a column of silica gel, eluting with petroleum ether and ethyl acetate (7:3). The solvent was removed under reduced pressure, leaving the white solid sulfoxide (Yield: 80%). A general reaction scheme is given in Fig. 4 ▸. X-ray-quality crystals were obtained from methanol solution by slow evaporation (m.p.: 421–423 K).

## Structure solution and step-by-step refinement overview

**Figure 5:**
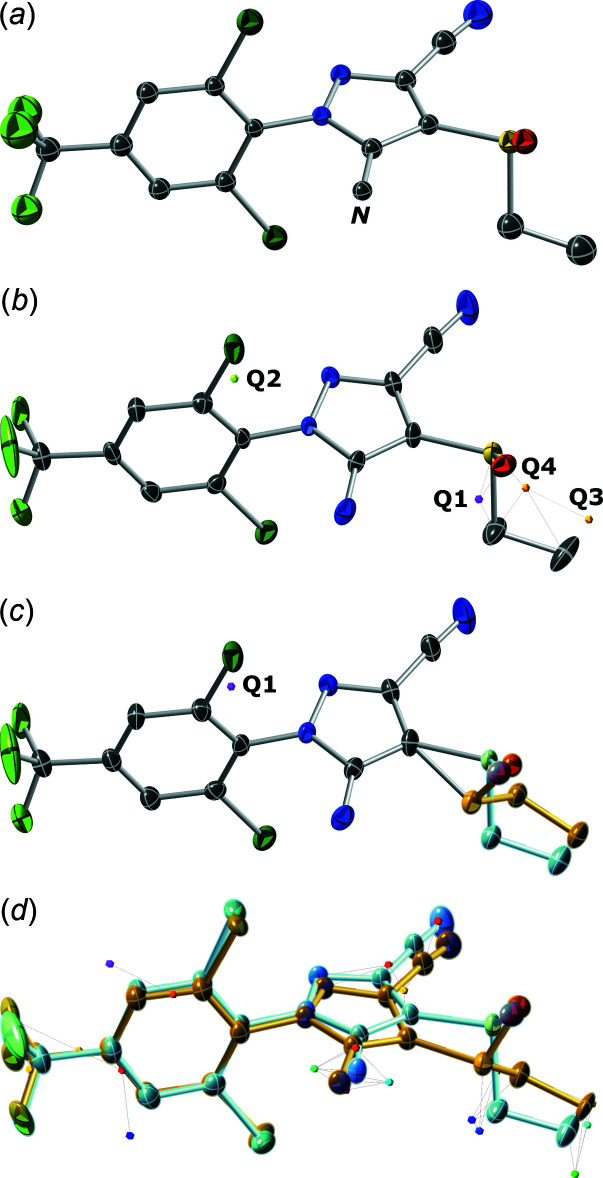
Snapshots of the structure model at various stages of complexity. (*a*) Initial model after structure solution by *SHELXT*. All atoms except the amine nitro­gen (here labelled *N*) were assigned correctly. (*b*) After anisotropic refinement, three of the largest difference map peaks (Q1,Q3,Q4) suggest disorder of the ethane­sulfinyl group. (*c*) A partial disorder model with the ethane­sulfinyl group split over two configurations reveals unrealistic distortion at the connection to the pyrazole ring, hinting that the disorder must extend further into the rest of the mol­ecule. (*d*) Modelling of whole-mol­ecule disorder satisfactorily accounts for all spurious electron density, revealing the major-component hydrogen atoms (small coloured dots).

In this section, the process of model building and refinement from an initial solution through to a final model incorporating whole-mol­ecule configurational disorder is described as a series of logical steps. A few snapshots of the model at each stage are given in Fig. 5 ▸, with corresponding refinement statistics summarized in Table 3 ▸. *SHELXL* RES files for each step are included in the supporting information.

*Step 1*: The structure solved quite easily using *SHELXT*, to give a starting model with all atom types assigned correctly apart from the amine nitro­gen, which had been tagged as a carbon. This starting model, depicted in Fig. 5 ▸ *a*, was readily corrected while assigning a sensible atom-numbering scheme to the model.

*Step 2*: Upon refinement of anisotropic displacement parameters (ADPs), the model looked quite reasonable. One fluorine ellipsoid is elongated (Fig. 5 ▸ *b*), but disorder of CF_3_ groups is common and easy to model. The difference map, however, revealed a few substantial electron-density peaks. In Fig. 5 ▸ *b*, the largest peak (labelled Q1) corresponded to 4.85 e Å^−3^, which is far too big to be ignored. The next three largest, labelled Q2, Q3, and Q4 ranged from 1.64–1.05 e Å^−3^. By inspection, a disorder model with a minor component for the ethane­sulfinyl substituent comprising Q1 (as S1′), O1 (copied as O1′) and Q4/Q3 (as C5′/C6′) looks plausible, but inverts the stereochemistry at the sulfur atom, thereby dictating configurational disorder. In the subsequent model, the major and minor components were assigned separate PARTs in *SHELXL*, occupancies were constrained to sum to unity *via* an FVAR (‘free variable’) parameter, and similarity restraints on geometry (SAME) and ADPs (SIMU) were added.

*Step 3*: Refinement of partial ethane­sulfinyl disorder gave the model depicted in Fig. 5 ▸ *c*. Here, the largest difference map peak (Q1) is now 1.44 e Å^−3^ and only about 0.8 Å from Cl1. A further problem with this partial disorder model is that the geometry of the bonding of the minor ethane­sulfinyl group to the pyrazole ring is severely distorted. In order to fix this, a second component for the pyrazole ring and consequently, much of the rest of the mol­ecule would be required. Thus, a model for whole-mol­ecule disorder was constructed by simply copying the previously non-disordered (‘PART 0’) atoms into the major and minor PARTs and tying the occupancies using the same FVAR parameter. The similarity (SAME and SIMU) restraints were strengthened (assigned a smaller effective uncertainty) so as to ensure that the minor-component bond distances and angles conform to those of the major.

*Step 4*: The resulting initial refinement of whole-mol­ecule disorder gave the model shown in Fig. 5 ▸ *d*. The previous largest difference map peak is now accounted for by the minor component Cl1′, such that the largest peaks now all correspond to hydrogen atoms.

*Step 5*: For the final refinement stage, hydrogen atoms were added and the constraints/restraints were optimized. For this structure, many of the disordered atom pairs are in very close proximity, so most were constrained using the *SHELXL* command EADP. For the minor ethane­sulfinyl group, the ADPs were restrained using RIGU (Thorn *et al.*, 2012 ▸). The tri­fluoro­methyl group adds an additional complication because the available electron density is not quite compatible with the occupancy factors refined for the main disorder model. For this reason, a separate occupancy for the fluorine atoms (but not the carbon) was set, which refined to 0.61 (4) for the major component. This is an approximate treatment, but wholly satisfactory in this case. Construction of a more sophisticated four-component disorder model is possible, but ultimately of limited scientific value. RIGU restraints were also sufficient to keep the minor CF_3_ group ADPs in check. The particular combination of constraints and restraints is largely dependent upon the nature of the disorder, and so should be decided on a case-by-case basis. Experimentation with different approaches provides a valuable learning opportunity. Overlap of disorder components in the final model is shown in Fig. 2 ▸. The main take-away message here is that construction of sensible disorder models can be straightforward and logical, but the essential criterion is that the model *must* make good chemical and crystallographic sense. Crystal-structure refinement requires more than an uncritical quest for low *R*-values.

A summary of crystal data, data collection, and refinement details is given in Table 4 ▸. All major-component hydrogen atoms were found in difference maps and all carbon-bound hydrogens were refined using riding models with constrained distances set to 0.95 Å (C*sp* ^2^—H), 0.98 Å (*R*CH_3_), 0.99 Å (*R*_2_CH_2_). The major-component NH_2_ hydrogen coordinates refined in a stable manner, but those of the minor component used a riding model with N—H distances set to 0.88 Å. *U*_iso_(H) parameters were assigned values of either 1.2*U*_eq_ or 1.5*U*_eq_ (*R*CH_3_ only) of the attached atom.

## Supplementary Material

Crystal structure: contains datablock(s) I, global. DOI: 10.1107/S205698902300035X/dj2062sup1.cif

Structure factors: contains datablock(s) I. DOI: 10.1107/S205698902300035X/dj2062Isup2.hkl

Intermediate models (RES files disguised as TXT) for the five steps given in the main manuscript, also the last checkCIF report for the revised CIF. DOI: 10.1107/S205698902300035X/dj2062sup3.txt

Supporting information file. DOI: 10.1107/S205698902300035X/dj2062sup4.txt

Supporting information file. DOI: 10.1107/S205698902300035X/dj2062sup5.txt

Supporting information file. DOI: 10.1107/S205698902300035X/dj2062sup6.txt

Supporting information file. DOI: 10.1107/S205698902300035X/dj2062sup7.txt

Supporting information file. DOI: 10.1107/S205698902300035X/dj2062sup8.pdf

Supporting information file. DOI: 10.1107/S205698902300035X/dj2062Isup9.cml

CCDC reference: 2235968

Additional supporting information:  crystallographic information; 3D view; checkCIF report

## Figures and Tables

**Table 1:** Selected torsion angles (°) in ethiprole Standard uncertainties for the minor component are much larger than for the major component as a result of its much lower occupancy factor.

Major	Torsion	Minor	Torsion
C1—N1—C7—C8	107.1 (7)	C1′—N1′—C7′—C8′	107 (4)
C9—C10—C13—F1	73.7 (10)	C9′—C10′—C13′—F1′	105 (3)
C1—C2—S1—O1	−45.6 (4)	C1′—C2′—S1′—O1′	−106 (3)

**Table 2:** Hydrogen-bond geometry (Å, °)

*D*—H⋯*A*	*D*—H	H⋯*A*	*D*⋯*A*	*D*—H⋯*A*
N3—H3*NA*⋯O1^i^	0.85 (2)	1.99 (2)	2.820 (6)	164 (3)
N3—H3*NB*⋯N4^ii^	0.85 (2)	2.31 (2)	3.150 (4)	172 (3)
C9—H9⋯O1^iii^	0.95	2.21	3.138 (6)	165
N3′—H3*ND*⋯O1′^i^	0.88	1.94	2.81 (5)	167
N3′—H3*NC*⋯N4′^ii^	0.88	2.10	2.87 (3)	145
C9′—H9′⋯O1′^iii^	0.95	2.31	3.17 (4)	151

Symmetry codes: (i) 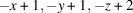 ; (ii) 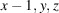 ; (iii) 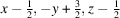 .

**Table 3:** Statistics for inter­mediate and final model building and refinement stages Steps 1–4 in the table correspond to the sequential snapshots shown in Fig. 5 ▸. The result of step 5 is shown in Fig. 2 ▸.

Step	*R*_1_ (%)	*wR*_2_ (%)	Δρ_max_ (e Å^−3^)	Δρ_min_ (e Å^−3^)
1	16.49	49.05	4.73	−2.23
2	9.22	32.70	4.87	−1.16
3	6.73	25.49	1.40	−1.07
4	5.51	21.73	0.81	−0.65
5	3.65	7.65	0.37	−0.25

**Table 4:** Experimental details

Crystal data	
Chemical formula	C_13_H_9_Cl_2_F_3_N_4_OS
*M*_r_	397.20
Crystal system, space group	Monoclinic, *P*2_1_/*n*
Temperature (K)	90
*a*, *b*, *c* (Å)	8.6199 (3), 12.7967 (5), 14.9178 (5)
β (°)	91.280 (1)
*V* (Å^3^)	1645.12 (10)
*Z*	4
Radiation type	Mo *K*α
μ (mm^−1^)	0.56
Crystal size (mm)	0.27 × 0.13 × 0.07

Data collection	
Diffractometer	Bruker D8 Venture dual source
Absorption correction	Multi-scan (*SADABS*; Krause *et al.*, 2015 ▸)
*T*_min_, *T*_max_	0.856, 0.971
No. of measured, independent and observed [*I* > 2σ(*I*)] reflections	23236, 3792, 3437
*R*_int_	0.031
(sin θ/λ)_max_ (Å^−1^)	0.651

Refinement	
*R*[*F* ^2^ > 2σ(*F* ^2^)], *wR*(*F* ^2^), *S*	0.037, 0.077, 1.26
No. of reflections	3792
No. of parameters	341
No. of restraints	108
H-atom treatment	H atoms treated by a mixture of independent and constrained refinement
Δρ_max_, Δρ_min_ (e Å^−3^)	0.37, −0.25

Computer programs: *APEX3* (Bruker, 2016 ▸), *SHELXT* (Sheldrick, 2015*a* ▸), *SHELXL2019/2* (Sheldrick, 2015*b* ▸), *XP* in *SHELXTL* (Sheldrick, 2008 ▸), *SHELX* (Sheldrick, 2008 ▸) and *publCIF* (Westrip, 2010 ▸).

## supplementary crystallographic information

### Crystal data

**Table:**

C_13_H_9_Cl_2_F_3_N_4_OS	*F*(000) = 800
*M**_r_* = 397.20	*D*_x_ = 1.604 Mg m^−^^3^
Monoclinic, *P*2_1_/*n*	Mo *K*α radiation, λ = 0.71073 Å
*a* = 8.6199 (3) Å	Cell parameters from 9978 reflections
*b* = 12.7967 (5) Å	θ = 2.7–27.5°
*c* = 14.9178 (5) Å	µ = 0.56 mm^−^^1^
β = 91.280 (1)°	*T* = 90 K
*V* = 1645.12 (10) Å^3^	Tablet, colourless
*Z* = 4	0.27 × 0.13 × 0.07 mm

### Data collection

**Table:**

Bruker D8 Venture dual source diffractometer	3792 independent reflections
Radiation source: microsource	3437 reflections with *I* > 2σ(*I*)
Detector resolution: 7.41 pixels mm^-1^	*R*_int_ = 0.031
φ and ω scans	θ_max_ = 27.6°, θ_min_ = 2.1°
Absorption correction: multi-scan (*SADABS*; Krause *et al.*, 2015)	*h* = −11→11
*T*_min_ = 0.856, *T*_max_ = 0.971	*k* = −15→16
23236 measured reflections	*l* = −19→19

### Refinement

**Table:**

Refinement on *F*^2^	Secondary atom site location: difference Fourier map
Least-squares matrix: full	Hydrogen site location: mixed
*R*[*F*^2^ > 2σ(*F*^2^)] = 0.037	H atoms treated by a mixture of independent and constrained refinement
*wR*(*F*^2^) = 0.077	*w* = 1/[σ^2^(*F*_o_^2^) + (0.0083*P*)^2^ + 1.5062*P*] where *P* = (*F*_o_^2^ + 2*F*_c_^2^)/3
*S* = 1.26	(Δ/σ)_max_ = 0.001
3792 reflections	Δρ_max_ = 0.37 e Å^−^^3^
341 parameters	Δρ_min_ = −0.25 e Å^−^^3^
108 restraints	Extinction correction: SHELXL-2019/2 (Sheldrick 2015b), Fc^*^=kFc[1+0.001xFc^2^λ^3^/sin(2θ)]^-1/4^
Primary atom site location: structure-invariant direct methods	Extinction coefficient: 0.0013 (3)

### Special details

**Table:**

Experimental. The crystal was mounted using polyisobutene oil on the tip of a fine glass fibre, which was fastened in a copper mounting pin with electrical solder. It was placed directly into the cold gas stream of a liquid-nitrogen based cryostat (Hope, 1994; Parkin & Hope, 1998). Diffraction data were collected with the crystal at 90K, which is standard practice in this laboratory for the majority of flash-cooled crystals.
Geometry. All esds (except the esd in the dihedral angle between two l.s. planes) are estimated using the full covariance matrix. The cell esds are taken into account individually in the estimation of esds in distances, angles and torsion angles; correlations between esds in cell parameters are only used when they are defined by crystal symmetry. An approximate (isotropic) treatment of cell esds is used for estimating esds involving l.s. planes.
Refinement. Refinement progress was checked using *Platon* (Spek, 2020) and by an *R*-tensor (Parkin, 2000). The final model was further checked with the IUCr utility *checkCIF*.

### Fractional atomic coordinates and isotropic or equivalent isotropic displacement parameters (Å^2^)

**Table:**

	*x*	*y*	*z*	*U*_iso_*/*U*_eq_	Occ. (<1)
N1	0.5445 (4)	0.5998 (6)	0.7093 (3)	0.0182 (5)	0.8670 (18)
N2	0.6936 (3)	0.6209 (5)	0.6832 (2)	0.0196 (5)	0.8670 (18)
N3	0.4028 (2)	0.5309 (3)	0.82962 (18)	0.0230 (5)	0.8670 (18)
H3NA	0.396 (3)	0.512 (2)	0.8842 (14)	0.028*	0.8670 (18)
H3NB	0.320 (3)	0.552 (2)	0.8046 (18)	0.028*	0.8670 (18)
N4	1.0770 (3)	0.6082 (3)	0.75630 (19)	0.0389 (7)	0.8670 (18)
C1	0.5370 (3)	0.5576 (3)	0.7931 (2)	0.0171 (5)	0.8670 (18)
C2	0.6907 (3)	0.5514 (2)	0.82449 (16)	0.0173 (5)	0.8670 (18)
C3	0.7781 (3)	0.5921 (2)	0.75424 (16)	0.0174 (5)	0.8670 (18)
C4	0.9451 (3)	0.6026 (3)	0.75381 (18)	0.0254 (6)	0.8670 (18)
S1	0.76917 (6)	0.50533 (5)	0.92572 (3)	0.01963 (17)	0.8670 (18)
O1	0.6751 (7)	0.5486 (3)	1.0009 (3)	0.0260 (8)	0.8670 (18)
C5	0.7180 (3)	0.36919 (18)	0.91758 (16)	0.0272 (5)	0.8670 (18)
H5A	0.604079	0.361134	0.921067	0.033*	0.8670 (18)
H5B	0.751054	0.340844	0.859280	0.033*	0.8670 (18)
C6	0.7973 (4)	0.3095 (3)	0.9937 (2)	0.0364 (7)	0.8670 (18)
H6A	0.909024	0.323806	0.993807	0.055*	0.8670 (18)
H6B	0.779664	0.234421	0.985451	0.055*	0.8670 (18)
H6C	0.754503	0.331730	1.050923	0.055*	0.8670 (18)
C7	0.4240 (4)	0.6102 (3)	0.6436 (2)	0.0168 (6)	0.8670 (18)
C8	0.3596 (4)	0.7075 (2)	0.6246 (2)	0.0186 (6)	0.8670 (18)
Cl1	0.41063 (10)	0.81431 (6)	0.68914 (5)	0.02727 (17)	0.8670 (18)
C9	0.2533 (5)	0.7197 (4)	0.5541 (3)	0.0234 (6)	0.8670 (18)
H9	0.210998	0.786461	0.540059	0.028*	0.8670 (18)
C10	0.2102 (8)	0.6321 (4)	0.5045 (3)	0.0228 (5)	0.8670 (18)
C11	0.2662 (8)	0.5337 (4)	0.5248 (4)	0.0226 (9)	0.8670 (18)
H11	0.233081	0.474522	0.491027	0.027*	0.8670 (18)
C12	0.372 (1)	0.5234 (4)	0.5956 (5)	0.0203 (6)	0.8670 (18)
Cl2	0.4408 (3)	0.4005 (3)	0.6233 (3)	0.0286 (4)	0.8670 (18)
C13	0.1079 (4)	0.6471 (3)	0.4223 (3)	0.0295 (5)	0.8670 (18)
F1	0.1853 (13)	0.6876 (12)	0.3572 (8)	0.056 (2)	0.61 (4)
F2	−0.0122 (15)	0.7092 (14)	0.4396 (10)	0.066 (3)	0.61 (4)
F3	0.0463 (14)	0.5561 (8)	0.3945 (8)	0.0377 (15)	0.61 (4)
N1'	0.527 (3)	0.594 (4)	0.7149 (19)	0.0182 (5)	0.1330 (18)
N2'	0.680 (2)	0.610 (4)	0.6972 (18)	0.0196 (5)	0.1330 (18)
N3'	0.359 (2)	0.522 (2)	0.8202 (16)	0.0230 (5)	0.1330 (18)
H3NC	0.279354	0.534504	0.784121	0.028*	0.1330 (18)
H3ND	0.345134	0.491878	0.872482	0.028*	0.1330 (18)
N4'	1.049 (2)	0.589 (3)	0.7759 (16)	0.0389 (7)	0.1330 (18)
C1'	0.501 (2)	0.548 (3)	0.7958 (17)	0.0171 (5)	0.1330 (18)
C2'	0.6496 (17)	0.5301 (16)	0.8317 (11)	0.0173 (5)	0.1330 (18)
C3'	0.7518 (17)	0.5701 (17)	0.7724 (13)	0.0174 (5)	0.1330 (18)
C4'	0.9195 (18)	0.582 (2)	0.7715 (16)	0.0254 (6)	0.1330 (18)
S1'	0.6671 (4)	0.4649 (3)	0.9351 (2)	0.0217 (11)	0.1330 (18)
O1'	0.712 (5)	0.545 (3)	1.006 (2)	0.034 (7)	0.1330 (18)
C5'	0.844 (2)	0.3953 (15)	0.9178 (10)	0.041 (4)	0.1330 (18)
H5'1	0.833948	0.350034	0.864145	0.049*	0.1330 (18)
H5'2	0.930407	0.445072	0.908355	0.049*	0.1330 (18)
C6'	0.878 (3)	0.329 (2)	1.0011 (15)	0.046 (6)	0.1330 (18)
H6'1	0.974518	0.289973	0.993367	0.069*	0.1330 (18)
H6'2	0.888340	0.374509	1.053665	0.069*	0.1330 (18)
H6'3	0.792253	0.279836	1.009625	0.069*	0.1330 (18)
C7'	0.404 (3)	0.6039 (18)	0.6499 (16)	0.0168 (6)	0.1330 (18)
C8'	0.334 (3)	0.6994 (16)	0.6320 (15)	0.0186 (6)	0.1330 (18)
Cl1'	0.3635 (7)	0.7996 (5)	0.7076 (4)	0.02727 (17)	0.1330 (18)
C9'	0.240 (3)	0.715 (2)	0.5570 (17)	0.0234 (6)	0.1330 (18)
H9'	0.192939	0.780913	0.545476	0.028*	0.1330 (18)
C10'	0.214 (5)	0.631 (2)	0.499 (2)	0.0228 (5)	0.1330 (18)
C11'	0.277 (6)	0.534 (3)	0.517 (3)	0.0226 (9)	0.1330 (18)
H11'	0.253091	0.476032	0.479105	0.027*	0.1330 (18)
C12'	0.376 (7)	0.522 (2)	0.591 (3)	0.0203 (6)	0.1330 (18)
Cl2'	0.461 (3)	0.4036 (19)	0.619 (2)	0.0286 (4)	0.1330 (18)
C13'	0.114 (2)	0.6495 (17)	0.4167 (14)	0.0295 (5)	0.1330 (18)
F1'	0.1932 (13)	0.6559 (17)	0.3441 (8)	0.044 (3)	0.39 (4)
F2'	0.028 (2)	0.7355 (9)	0.4227 (9)	0.044 (2)	0.39 (4)
F3'	0.012 (2)	0.5720 (13)	0.4029 (13)	0.038 (3)	0.39 (4)

### Atomic displacement parameters (Å^2^)

**Table:**

	*U*^11^	*U*^22^	*U*^33^	*U*^12^	*U*^13^	*U*^23^
N1	0.0171 (11)	0.0231 (12)	0.0145 (9)	−0.0004 (12)	0.0000 (9)	0.0037 (8)
N2	0.0165 (9)	0.0266 (18)	0.0159 (15)	−0.0032 (9)	0.0013 (9)	0.0046 (11)
N3	0.0154 (13)	0.0370 (13)	0.0167 (10)	−0.0001 (12)	−0.0010 (11)	0.0069 (9)
N4	0.0218 (12)	0.067 (2)	0.0278 (16)	−0.0038 (12)	−0.0001 (10)	0.0073 (13)
C1	0.0185 (14)	0.0194 (14)	0.0133 (9)	0.0001 (14)	0.0009 (11)	0.0016 (8)
C2	0.0158 (12)	0.0227 (14)	0.0134 (9)	0.0001 (9)	0.0001 (10)	0.0019 (9)
C3	0.0159 (11)	0.0210 (15)	0.0153 (13)	−0.0015 (9)	0.0000 (9)	0.0028 (9)
C4	0.0219 (13)	0.0375 (19)	0.0169 (15)	−0.0041 (11)	−0.0009 (9)	0.0056 (11)
S1	0.0170 (3)	0.0269 (3)	0.0149 (2)	−0.0014 (2)	−0.00284 (18)	0.0052 (2)
O1	0.038 (3)	0.0251 (13)	0.0155 (11)	−0.0019 (12)	0.0043 (11)	0.0007 (9)
C5	0.0363 (14)	0.0218 (12)	0.0234 (11)	0.0061 (10)	−0.0017 (10)	0.0010 (9)
C6	0.054 (2)	0.0304 (15)	0.0248 (14)	0.0151 (16)	0.0026 (14)	0.0066 (11)
C7	0.0147 (13)	0.0246 (11)	0.0112 (10)	−0.0020 (9)	0.0023 (9)	0.0034 (8)
C8	0.0182 (15)	0.0207 (11)	0.0170 (11)	−0.0018 (9)	0.0002 (9)	−0.0008 (9)
Cl1	0.0333 (4)	0.0225 (3)	0.0256 (4)	0.0016 (3)	−0.0092 (3)	−0.0049 (3)
C9	0.0221 (13)	0.0256 (12)	0.0221 (10)	0.0039 (10)	−0.0042 (9)	0.0016 (9)
C10	0.0186 (10)	0.0303 (11)	0.0194 (11)	−0.0013 (8)	−0.0032 (9)	0.0002 (9)
C11	0.0230 (16)	0.0252 (10)	0.0195 (15)	−0.0047 (9)	−0.0010 (14)	−0.0030 (9)
C12	0.0211 (10)	0.0209 (10)	0.0189 (12)	0.0001 (8)	0.0036 (10)	0.0033 (8)
Cl2	0.0366 (10)	0.0199 (3)	0.0290 (6)	0.0021 (6)	−0.0037 (7)	0.0017 (3)
C13	0.0243 (11)	0.0371 (13)	0.0269 (12)	0.0011 (9)	−0.0082 (9)	−0.0025 (10)
F1	0.061 (3)	0.075 (5)	0.031 (3)	−0.027 (3)	−0.025 (2)	0.028 (3)
F2	0.047 (4)	0.084 (6)	0.066 (4)	0.037 (4)	−0.036 (3)	−0.033 (4)
F3	0.032 (3)	0.046 (3)	0.034 (2)	−0.002 (2)	−0.017 (2)	−0.0078 (18)
N1'	0.0171 (11)	0.0231 (12)	0.0145 (9)	−0.0004 (12)	0.0000 (9)	0.0037 (8)
N2'	0.0165 (9)	0.0266 (18)	0.0159 (15)	−0.0032 (9)	0.0013 (9)	0.0046 (11)
N3'	0.0154 (13)	0.0370 (13)	0.0167 (10)	−0.0001 (12)	−0.0010 (11)	0.0069 (9)
N4'	0.0218 (12)	0.067 (2)	0.0278 (16)	−0.0038 (12)	−0.0001 (10)	0.0073 (13)
C1'	0.0185 (14)	0.0194 (14)	0.0133 (9)	0.0001 (14)	0.0009 (11)	0.0016 (8)
C2'	0.0158 (12)	0.0227 (14)	0.0134 (9)	0.0001 (9)	0.0001 (10)	0.0019 (9)
C3'	0.0159 (11)	0.0210 (15)	0.0153 (13)	−0.0015 (9)	0.0000 (9)	0.0028 (9)
C4'	0.0219 (13)	0.0375 (19)	0.0169 (15)	−0.0041 (11)	−0.0009 (9)	0.0056 (11)
S1'	0.020 (2)	0.026 (2)	0.0183 (17)	−0.0026 (15)	−0.0009 (13)	0.0052 (14)
O1'	0.033 (15)	0.038 (9)	0.030 (7)	0.005 (7)	−0.006 (6)	−0.004 (6)
C5'	0.048 (9)	0.048 (9)	0.026 (7)	0.018 (8)	0.005 (6)	0.013 (6)
C6'	0.046 (11)	0.061 (14)	0.032 (9)	0.019 (11)	0.008 (9)	0.027 (10)
C7'	0.0147 (13)	0.0246 (11)	0.0112 (10)	−0.0020 (9)	0.0023 (9)	0.0034 (8)
C8'	0.0182 (15)	0.0207 (11)	0.0170 (11)	−0.0018 (9)	0.0002 (9)	−0.0008 (9)
Cl1'	0.0333 (4)	0.0225 (3)	0.0256 (4)	0.0016 (3)	−0.0092 (3)	−0.0049 (3)
C9'	0.0221 (13)	0.0256 (12)	0.0221 (10)	0.0039 (10)	−0.0042 (9)	0.0016 (9)
C10'	0.0186 (10)	0.0303 (11)	0.0194 (11)	−0.0013 (8)	−0.0032 (9)	0.0002 (9)
C11'	0.0230 (16)	0.0252 (10)	0.0195 (15)	−0.0047 (9)	−0.0010 (14)	−0.0030 (9)
C12'	0.0211 (10)	0.0209 (10)	0.0189 (12)	0.0001 (8)	0.0036 (10)	0.0033 (8)
Cl2'	0.0366 (10)	0.0199 (3)	0.0290 (6)	0.0021 (6)	−0.0037 (7)	0.0017 (3)
C13'	0.0243 (11)	0.0371 (13)	0.0269 (12)	0.0011 (9)	−0.0082 (9)	−0.0025 (10)
F1'	0.025 (3)	0.090 (8)	0.017 (2)	−0.002 (3)	−0.005 (2)	0.008 (3)
F2'	0.048 (5)	0.043 (4)	0.041 (4)	0.018 (3)	−0.030 (3)	−0.008 (3)
F3'	0.029 (5)	0.044 (4)	0.041 (5)	−0.012 (4)	−0.019 (3)	0.007 (3)

### Geometric parameters (Å, º)

**Table:**

N1—C1	1.364 (3)	N1'—C1'	1.369 (16)
N1—N2	1.378 (3)	N1'—N2'	1.369 (16)
N1—C7	1.419 (3)	N1'—C7'	1.428 (15)
N2—C3	1.325 (3)	N2'—C3'	1.368 (16)
N3—C1	1.335 (3)	N3'—C1'	1.323 (16)
N3—H3NA	0.85 (2)	N3'—H3NC	0.8800
N3—H3NB	0.85 (2)	N3'—H3ND	0.8800
N4—C4	1.139 (3)	N4'—C4'	1.123 (16)
C1—C2	1.398 (3)	C1'—C2'	1.397 (15)
C2—C3	1.404 (3)	C2'—C3'	1.362 (14)
C2—S1	1.743 (2)	C2'—S1'	1.757 (13)
C3—C4	1.446 (3)	C3'—C4'	1.455 (15)
S1—O1	1.504 (4)	S1'—O1'	1.519 (18)
S1—C5	1.801 (3)	S1'—C5'	1.793 (13)
C5—C6	1.518 (3)	C5'—C6'	1.527 (16)
C5—H5A	0.9900	C5'—H5'1	0.9900
C5—H5B	0.9900	C5'—H5'2	0.9900
C6—H6A	0.9800	C6'—H6'1	0.9800
C6—H6B	0.9800	C6'—H6'2	0.9800
C6—H6C	0.9800	C6'—H6'3	0.9800
C7—C8	1.389 (3)	C7'—C8'	1.383 (16)
C7—C12	1.391 (4)	C7'—C12'	1.392 (16)
C8—C9	1.388 (3)	C8'—C9'	1.385 (16)
C8—Cl1	1.724 (3)	C8'—Cl1'	1.723 (16)
C9—C10	1.389 (4)	C9'—C10'	1.391 (16)
C9—H9	0.9500	C9'—H9'	0.9500
C10—C11	1.379 (4)	C10'—C11'	1.382 (16)
C10—C13	1.507 (3)	C10'—C13'	1.503 (15)
C11—C12	1.386 (4)	C11'—C12'	1.384 (16)
C11—H11	0.9500	C11'—H11'	0.9500
C12—Cl2	1.728 (3)	C12'—Cl2'	1.727 (16)
C13—F1	1.299 (8)	C13'—F1'	1.294 (18)
C13—F2	1.334 (6)	C13'—F2'	1.333 (17)
C13—F3	1.342 (7)	C13'—F3'	1.338 (17)
			
C1—N1—N2	113.6 (2)	C1'—N1'—N2'	114.4 (15)
C1—N1—C7	128.4 (3)	C1'—N1'—C7'	120.2 (18)
N2—N1—C7	117.4 (3)	N2'—N1'—C7'	124.2 (19)
C3—N2—N1	102.6 (2)	C3'—N2'—N1'	101.8 (14)
C1—N3—H3NA	122.6 (18)	C1'—N3'—H3NC	120.0
C1—N3—H3NB	118 (2)	C1'—N3'—H3ND	120.0
H3NA—N3—H3NB	116 (3)	H3NC—N3'—H3ND	120.0
N3—C1—N1	122.3 (2)	N3'—C1'—N1'	121.5 (17)
N3—C1—C2	132.3 (2)	N3'—C1'—C2'	134.3 (17)
N1—C1—C2	105.4 (2)	N1'—C1'—C2'	104.0 (13)
C1—C2—C3	104.42 (19)	C3'—C2'—C1'	106.9 (12)
C1—C2—S1	130.98 (19)	C3'—C2'—S1'	134.8 (12)
C3—C2—S1	124.60 (17)	C1'—C2'—S1'	118.2 (12)
N2—C3—C2	113.9 (2)	C2'—C3'—N2'	112.8 (13)
N2—C3—C4	120.0 (2)	C2'—C3'—C4'	134.7 (15)
C2—C3—C4	126.1 (2)	N2'—C3'—C4'	112.4 (15)
N4—C4—C3	177.2 (3)	N4'—C4'—C3'	176 (2)
O1—S1—C2	108.4 (2)	O1'—S1'—C2'	107.9 (17)
O1—S1—C5	105.8 (2)	O1'—S1'—C5'	103.5 (15)
C2—S1—C5	100.32 (12)	C2'—S1'—C5'	99.6 (8)
C6—C5—S1	109.32 (19)	C6'—C5'—S1'	107.8 (12)
C6—C5—H5A	109.8	C6'—C5'—H5'1	110.2
S1—C5—H5A	109.8	S1'—C5'—H5'1	110.2
C6—C5—H5B	109.8	C6'—C5'—H5'2	110.2
S1—C5—H5B	109.8	S1'—C5'—H5'2	110.2
H5A—C5—H5B	108.3	H5'1—C5'—H5'2	108.5
C5—C6—H6A	109.5	C5'—C6'—H6'1	109.5
C5—C6—H6B	109.5	C5'—C6'—H6'2	109.5
H6A—C6—H6B	109.5	H6'1—C6'—H6'2	109.5
C5—C6—H6C	109.5	C5'—C6'—H6'3	109.5
H6A—C6—H6C	109.5	H6'1—C6'—H6'3	109.5
H6B—C6—H6C	109.5	H6'2—C6'—H6'3	109.5
C8—C7—C12	119.2 (3)	C8'—C7'—C12'	118.6 (15)
C8—C7—N1	120.6 (3)	C8'—C7'—N1'	121 (2)
C12—C7—N1	120.2 (3)	C12'—C7'—N1'	119 (2)
C9—C8—C7	120.7 (3)	C7'—C8'—C9'	121.8 (18)
C9—C8—Cl1	119.3 (3)	C7'—C8'—Cl1'	118.4 (16)
C7—C8—Cl1	120.0 (2)	C9'—C8'—Cl1'	119.8 (16)
C8—C9—C10	118.4 (3)	C8'—C9'—C10'	118.2 (19)
C8—C9—H9	120.8	C8'—C9'—H9'	120.9
C10—C9—H9	120.8	C10'—C9'—H9'	120.9
C11—C10—C9	122.2 (3)	C11'—C10'—C9'	121.3 (17)
C11—C10—C13	119.4 (3)	C11'—C10'—C13'	121.0 (18)
C9—C10—C13	118.3 (3)	C9'—C10'—C13'	117.7 (17)
C10—C11—C12	118.4 (4)	C10'—C11'—C12'	119 (2)
C10—C11—H11	120.8	C10'—C11'—H11'	120.5
C12—C11—H11	120.8	C12'—C11'—H11'	120.5
C11—C12—C7	121.0 (3)	C11'—C12'—C7'	120.9 (18)
C11—C12—Cl2	119.0 (3)	C11'—C12'—Cl2'	122.9 (18)
C7—C12—Cl2	119.9 (3)	C7'—C12'—Cl2'	116.1 (17)
F1—C13—F2	108.7 (6)	F1'—C13'—F2'	108.0 (16)
F1—C13—F3	108.8 (7)	F1'—C13'—F3'	105.8 (16)
F2—C13—F3	105.9 (5)	F2'—C13'—F3'	104.8 (15)
F1—C13—C10	111.0 (5)	F1'—C13'—C10'	113 (2)
F2—C13—C10	111.2 (5)	F2'—C13'—C10'	112.7 (17)
F3—C13—C10	111.1 (6)	F3'—C13'—C10'	111.8 (19)
			
C1—N1—N2—C3	−1.1 (8)	C1'—N1'—N2'—C3'	0 (6)
C7—N1—N2—C3	−172.6 (5)	C7'—N1'—N2'—C3'	−167 (4)
N2—N1—C1—N3	−179.3 (5)	N2'—N1'—C1'—N3'	−176 (4)
C7—N1—C1—N3	−8.9 (10)	C7'—N1'—C1'—N3'	−8 (7)
N2—N1—C1—C2	0.6 (8)	N2'—N1'—C1'—C2'	−1 (6)
C7—N1—C1—C2	171.0 (6)	C7'—N1'—C1'—C2'	167 (3)
N3—C1—C2—C3	180.0 (5)	N3'—C1'—C2'—C3'	176 (4)
N1—C1—C2—C3	0.1 (5)	N1'—C1'—C2'—C3'	2 (4)
N3—C1—C2—S1	0.1 (7)	N3'—C1'—C2'—S1'	−3 (6)
N1—C1—C2—S1	−179.8 (4)	N1'—C1'—C2'—S1'	−177 (3)
N1—N2—C3—C2	1.1 (6)	C1'—C2'—C3'—N2'	−2 (4)
N1—N2—C3—C4	−179.7 (5)	S1'—C2'—C3'—N2'	177 (3)
C1—C2—C3—N2	−0.8 (5)	C1'—C2'—C3'—C4'	176 (3)
S1—C2—C3—N2	179.0 (4)	S1'—C2'—C3'—C4'	−5 (5)
C1—C2—C3—C4	−180.0 (3)	N1'—N2'—C3'—C2'	1 (5)
S1—C2—C3—C4	−0.1 (5)	N1'—N2'—C3'—C4'	−177 (3)
C1—C2—S1—O1	−45.6 (4)	C3'—C2'—S1'—O1'	75 (3)
C3—C2—S1—O1	134.5 (3)	C1'—C2'—S1'—O1'	−106 (3)
C1—C2—S1—C5	64.9 (4)	C3'—C2'—S1'—C5'	−33 (3)
C3—C2—S1—C5	−114.9 (3)	C1'—C2'—S1'—C5'	146 (2)
O1—S1—C5—C6	−75.9 (3)	O1'—S1'—C5'—C6'	71 (2)
C2—S1—C5—C6	171.4 (2)	C2'—S1'—C5'—C6'	−178.0 (17)
C1—N1—C7—C8	107.1 (7)	C1'—N1'—C7'—C8'	107 (4)
N2—N1—C7—C8	−82.8 (7)	N2'—N1'—C7'—C8'	−86 (5)
C1—N1—C7—C12	−74.7 (8)	C1'—N1'—C7'—C12'	−87 (5)
N2—N1—C7—C12	95.4 (8)	N2'—N1'—C7'—C12'	80 (6)
C12—C7—C8—C9	−4.9 (5)	C12'—C7'—C8'—C9'	0 (3)
N1—C7—C8—C9	173.2 (3)	N1'—C7'—C8'—C9'	166 (2)
C12—C7—C8—Cl1	174.8 (5)	C12'—C7'—C8'—Cl1'	177 (3)
N1—C7—C8—Cl1	−7.1 (4)	N1'—C7'—C8'—Cl1'	−16 (2)
C7—C8—C9—C10	1.6 (5)	C7'—C8'—C9'—C10'	0 (2)
Cl1—C8—C9—C10	−178.1 (4)	Cl1'—C8'—C9'—C10'	−178 (3)
C8—C9—C10—C11	1.8 (8)	C8'—C9'—C10'—C11'	2 (5)
C8—C9—C10—C13	−173.8 (4)	C8'—C9'—C10'—C13'	−179 (3)
C9—C10—C11—C12	−1.7 (11)	C9'—C10'—C11'—C12'	−4 (8)
C13—C10—C11—C12	173.8 (7)	C13'—C10'—C11'—C12'	176 (5)
C10—C11—C12—C7	−1.8 (12)	C10'—C11'—C12'—C7'	4 (8)
C10—C11—C12—Cl2	178.8 (7)	C10'—C11'—C12'—Cl2'	−180 (5)
C8—C7—C12—C11	5.1 (10)	C8'—C7'—C12'—C11'	−2 (7)
N1—C7—C12—C11	−173.1 (7)	N1'—C7'—C12'—C11'	−168 (5)
C8—C7—C12—Cl2	−175.6 (5)	C8'—C7'—C12'—Cl2'	−178 (3)
N1—C7—C12—Cl2	6.3 (9)	N1'—C7'—C12'—Cl2'	15 (5)
C11—C10—C13—F1	−102.0 (11)	C11'—C10'—C13'—F1'	−76 (5)
C9—C10—C13—F1	73.7 (10)	C9'—C10'—C13'—F1'	105 (3)
C11—C10—C13—F2	136.9 (12)	C11'—C10'—C13'—F2'	161 (4)
C9—C10—C13—F2	−47.4 (12)	C9'—C10'—C13'—F2'	−18 (4)
C11—C10—C13—F3	19.2 (9)	C11'—C10'—C13'—F3'	44 (5)
C9—C10—C13—F3	−165.1 (7)	C9'—C10'—C13'—F3'	−136 (3)

### Hydrogen-bond geometry (Å, º)

**Table:**

*D*—H···*A*	*D*—H	H···*A*	*D*···*A*	*D*—H···*A*
N3—H3*NA*···O1^i^	0.85 (2)	1.99 (2)	2.820 (6)	164 (3)
N3—H3*NB*···N4^ii^	0.85 (2)	2.31 (2)	3.150 (4)	172 (3)
C9—H9···O1^iii^	0.95	2.21	3.138 (6)	165
N3′—H3*ND*···O1′^i^	0.88	1.94	2.81 (5)	167
N3′—H3*NC*···N4′^ii^	0.88	2.10	2.87 (3)	145
C9′—H9′···O1′^iii^	0.95	2.31	3.17 (4)	151

Symmetry codes: (i) −*x*+1, −*y*+1, −*z*+2; (ii) *x*−1, *y*, *z*; (iii) *x*−1/2, −*y*+3/2, *z*−1/2.
