# Supplementary material for: Crystal structure of the insecticide ethiprole (C13H9Cl2F3N4OS): a case study of whole-mol­ecule configurational disorder
Source: Acta Crystallogr E Crystallogr Commun. 2023 Jan 19;79(Pt 2):54–9. doi: 10.1107/S205698902300035X (PMC9912465; doi:10.1107/S205698902300035X)
Supplement: Supplementary file 8 [file e-79-00054-sup8.pdf]

checkCIF (basic structural check) running

Checking for embedded fcf data in CIF ...
Found embedded fcf data in CIF. Extracting fcf data from uploaded CIF, please wait . . .

checkCIF/PLATON (basic structural check)

Structure factors have been supplied for datablock(s) ethiprole

THIS REPORT IS FOR GUIDANCE ONLY. IF USED AS PART OF A REVIEW PROCEDURE FOR PUBLICATION, IT SHOULD NOT REPLACE THE EXPERTISE OF AN EXPERIENCED CRYSTALLOGRAPHIC REFEREE.

No syntax errors found. CIF dictionary
Please wait while processing .... Interpreting this report

Structure factor report

Datablock: ethiprole

Bond precision:	= 0.0000 A		Wavelength=0.71073
Cell:	a=8.6199(3)	b=12.7967(5)	c=14.9178(5)
	alpha=90	beta=91.280(1)	gamma=90
Temperature:	90 K		

	Calculated	Reported
Volume	1645.12(10)	1645.12(10)
Space group	P 21/n	P 21/n
Hall group	-P 2yn	-P 2yn
Moiety formula	C11.27 H7.80 Cl1.73 F1.83 N3.47 O0.87 S0.87, C1.73 H1.20 Cl0.27	C13 H9 Cl2 F3 N4 O S
Sum formula	C13 H9 Cl2 F3 N4 O S	C13 H9 Cl2 F3 N4 O S
Mr	397.20	397.20
Dx,g cm-3	1.604	1.604
Z	4	4
Mu (mm-1)	0.561	0.561
F000	800.0	800.0
F000'	802.07	
h,k,lmax	11,16,19	11,16,19
Nref	3800	3792
Tmin,Tmax	0.916,0.961	0.856,0.971
Tmin'	0.859	

Correction method= # Reported T Limits: Tmin=0.856 Tmax=0.971
AbsCorr = MULTI-SCAN

Data completeness= 0.998 Theta(max)= 27.549

R(reflections)= 0.0365(3437) wR2(reflections)= 0.0766(3792)

S = 1.258 Npar= 341

The following ALERTS were generated. Each ALERT has the format
test-name_ALERT_alert-type_alert-level.

Click on the hyperlinks for more details of the test.

Alert level C

PLAT042_ALERT_1_C Calc. and Reported MoietyFormula Strings Differ Please Check
PLAT906_ALERT_3_C Large K Value in the Analysis of Variance ...... 4.531 Check

Alert level G

PLAT002_ALERT_2_G Number of Distance or Angle Restraints on AtSite 50 Note
PLAT007_ALERT_5_G Number of Unrefined Donor-H Atoms .... 2 Report
PLAT171_ALERT_4_G The CIF-Embedded .res File Contains EADP Records 17 Report
PLAT174_ALERT_4_G The CIF-Embedded .res File Contains FLAT Records 1 Report
PLAT175_ALERT_4_G The CIF-Embedded .res File Contains SAME Records 1 Report
PLAT176_ALERT_4_G The CIF-Embedded .res File Contains SADI Records 1 Report
PLAT187_ALERT_4_G The CIF-Embedded .res File Contains RIGU Records 2 Report
PLAT301_ALERT_3_G Main Residue Disorder ....(Resd 1) 100% Note
PLAT302_ALERT_4_G Anion/Solvent/Minor-Residue Disorder (Resd 2) 100% Note
PLAT304_ALERT_4_G Non-Integer Number of Atoms in ..... (Resd 1) 27.84 Check
PLAT304_ALERT_4_G Non-Integer Number of Atoms in ..... (Resd 2) 5.16 Check
PLAT720_ALERT_4_G Number of Unusual/Non-Standard Labels .... 9 Note
PLAT860_ALERT_3_G Number of Least-Squares Restraints .... 108 Note
PLAT912_ALERT_4_G Missing # of FCF Reflections Above STh/L= 0.600 8 Note

0 **ALERT level A** = Most likely a serious problem - resolve or explain
0 **ALERT level B** = A potentially serious problem, consider carefully
2 **ALERT level C** = Check. Ensure it is not caused by an omission or oversight
14 **ALERT level G** = General information/check it is not something unexpected

1 ALERT type 1 CIF construction/syntax error, inconsistent or missing data
1 ALERT type 2 Indicator that the structure model may be wrong or deficient
3 ALERT type 3 Indicator that the structure quality may be low
10 ALERT type 4 Improvement, methodology, query or suggestion
1 ALERT type 5 Informative message, check

It is advisable to attempt to resolve as many as possible of the alerts in all categories. Often the minor alerts point to easily fixed oversights, errors and omissions in your CIF or refinement strategy, so attention to these fine details can be worthwhile. In order to resolve some of the more serious problems it may be necessary to carry out additional measurements or structure refinements. However, the purpose of your study may justify the reported deviations and the more serious of these should normally be commented upon in the discussion or experimental section of a paper or in the "special_details" fields of the CIF. checkCIF was carefully designed to identify outliers and unusual parameters, but every test has its limitations and alerts that are not important in a particular case may appear. Conversely, the absence of alerts does not guarantee there are no aspects of the results needing attention. It is up to the individual to critically assess their own results and, if necessary, seek expert advice.

Publication of your CIF in IUCr journals

A basic structural check has been run on your CIF. These basic checks will be run on all CIFs submitted for publication in IUCr journals (*Acta Crystallographica*, *Journal of Applied Crystallography*, *Journal of Synchrotron Radiation*); however, if you intend to submit to *Acta Crystallographica Section C* or *E* or *IUCrData*, you should make sure that full publication checks are run on the final version of your CIF prior to submission.

Publication of your CIF in other journals

Please refer to the *Notes for Authors* of the relevant journal for any special instructions relating to CIF submission.

PLATON version of 28/11/2022; check.def file version of 28/11/2022

Datablock ethiprole - ellipsoid plot

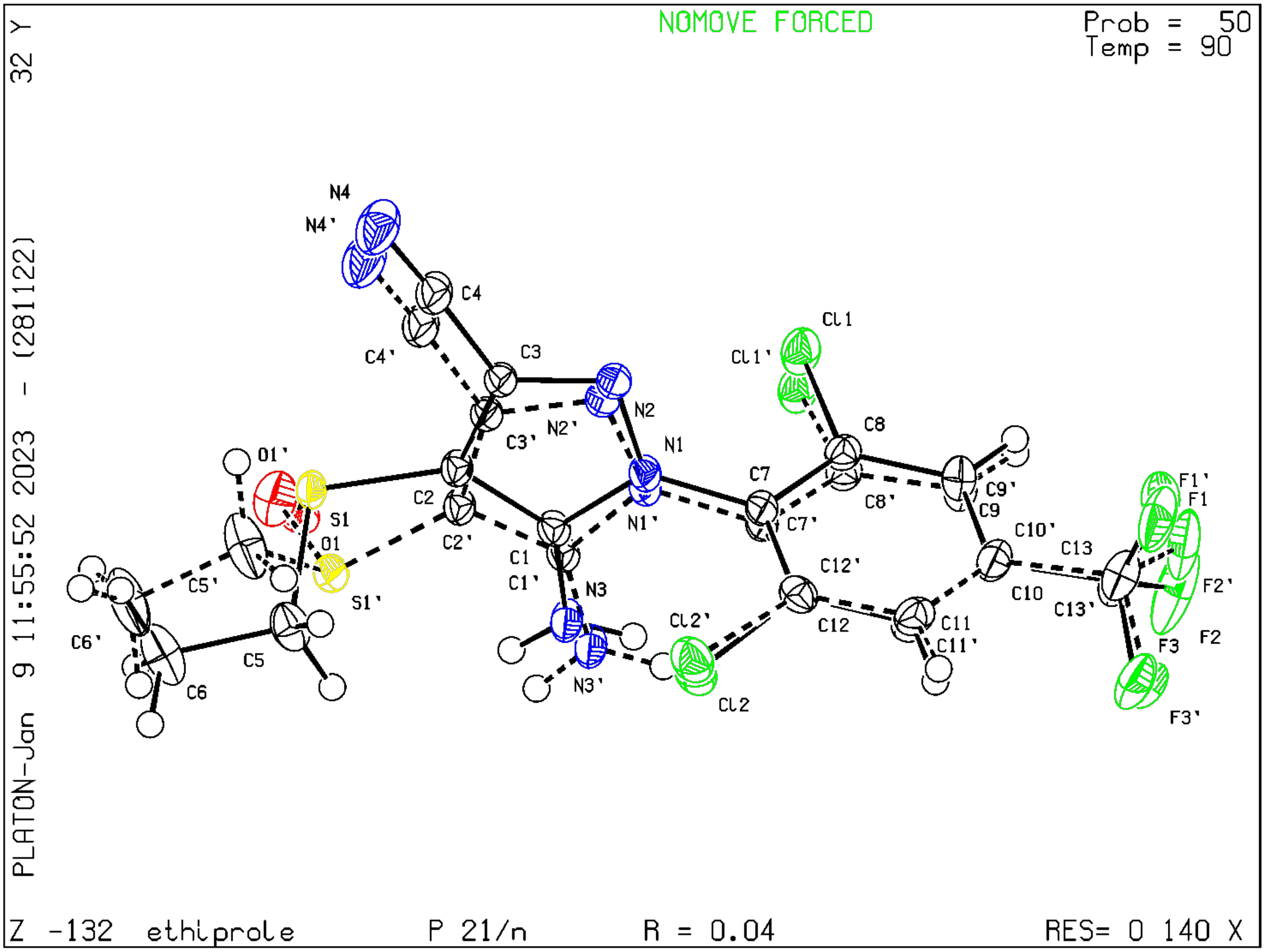

Download CIF editor (publCIF) from the IUCr
Download CIF editor (enCIFer) from the CCDC
Test a new CIF entry
